# Continuous flow enantioselective arylation of aldehydes with ArZnEt using triarylboroxins as the ultimate source of aryl groups

**DOI:** 10.3762/bjoc.5.56

**Published:** 2009-10-15

**Authors:** Julien Rolland, Xacobe C Cambeiro, Carles Rodríguez-Escrich, Miquel A Pericàs

**Affiliations:** 1 Institute of Chemical Research of Catalonia; Avinguda Països Catalans, 16; 43007 Tarragona, Spain; 2 Departament de Química Orgànica, Universitat de Barcelona; 08028 Barcelona, Spain

**Keywords:** asymmetric synthesis, continuous flow, diarylmethanols, solid-supported catalyst, triarylboroxins

## Abstract

A continuous flow system for the synthesis of enantioenriched diarylmethanols from aldehydes is described. The system uses an amino alcohol-functionalized polystyrene resin as the catalyst, and the arylating agent is conveniently prepared by transmetallation of triarylboroxins with diethylzinc.

## Introduction

**Figure 1:**
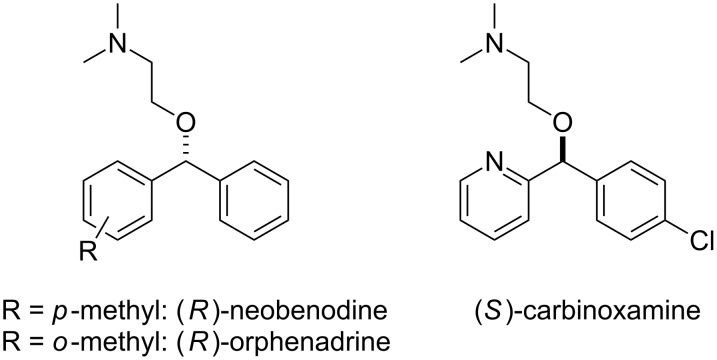
Biologically active diarylmethanol derivatives.

Diarylmethanols constitute the basic scaffold in several important drugs such as antihistamines and muscle relaxants (*R*)-neobenodine, (*R*)-orphenadrine or (*S*)-carbinoxamine (Figure 1) [1]. Despite the apparent simplicity of the structures, their asymmetric synthesis is not trivial. For instance, access to these structures through enantioselective reduction of the corresponding ketones can become troublesome when both aryl groups are similar in their electronic and steric properties [2–4].

On the other hand, enantioselective arylation of aldehydes with organozinc reagents appears as a most convenient alternative, since the initial aldehyde undergoing addition presents two very different groups (namely, a H atom and an aryl group) and hence offers good opportunities for enantiocontrol [5–7].

Whereas the catalytic enantioselective addition of diethylzinc to aldehydes has been thoroughly studied, progress in the control of the analogous asymmetric arylation has been hampered by the fact that Ar_2_Zn species are several orders of magnitude more active than their dialkyl counterparts [8]. In this way, when diphenylzinc has been used as the arylating species in these processes, the background, non catalyzed reaction gives rise to a racemic product, which significantly erodes the global enantioselectivity of the reactions [6,9].

**Figure 2:**
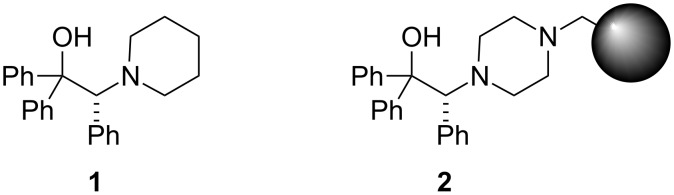
Structures of (*R*)-1,1,2-triphenyl-2-(piperidin-1-yl)ethanol (**1**) and its polystyrene-immobilized analogue **2**.

The most successful approach to overcome this difficulty comes from the Bolm laboratory and has been based on the use of the comparatively less reactive mixed species PhEtZn, easily prepared from a mixture of Ph_2_Zn and Et_2_Zn [10–13]. With this strategy, it has become possible to achieve good levels of enantioselectivity in the arylation reaction, although usually at the cost of low catalytic activity, high catalytic loadings being required for the achievement of satisfactory yields [14–15]. In contrast, β-amino alcohol **1** (Figure 2), developed in our laboratory [16], showed high activity and enantioselectivity in the ethylation [16], methylation [17], and arylation [18] of a wide family of substrates at low catalyst loadings.

In recent times, we have developed strategies for the immobilization of analogues of **1** onto solid supports [19–22]. Among the ligands resulting from these studies, the polystyrene-supported catalyst **2** displayed levels of catalytic activity and selectivity comparable to those of the homogeneous model **1**. Noteworthy, this catalytic resin has allowed the development of the first catalytic enantioselective arylation of aldehydes employing an insoluble catalyst [22], and has been used as the basis for a single-pass, continuous flow highly enantioselective ethylation of aldehydes characterized by very short residence times (down to 2.8 min) [23]. According to these precedents, we considered that **2** could be a good candidate for a planned continuous enantioselective production of diarylmethanols.

When a large scale production of diarylmethanols involving the enantioselective transfer of aryl groups from zinc to aldehydes is considered, the cost of the arylating agent becomes an important issue. From this perspective, substantial efforts have been devoted to improve the economy of this process, by replacing the expensive Ph_2_Zn by other, more convenient, aryl sources.

**Scheme 1:**
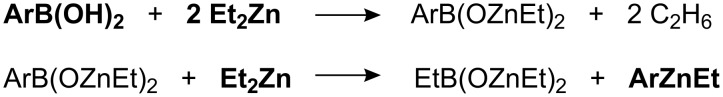
Generation of the mixed ArZnEt species from a boronic acid and Et_2_Zn.

To this end, the use of arylboron species has provided particularly good results. In contrast to what happens with diarylzinc reagents, a wide variety of arylboronic acids are commercially available at a convenient price, and these species have been explored as the ultimate source of aryl groups [24–29]. In these approaches generally good results have been obtained, but at the expense of using a large excess of diethylzinc for the transmetallation step, since two non-productive equivalents of the reagent are consumed in the initial reaction with the boronic acid (Scheme 1).

**Scheme 2:**
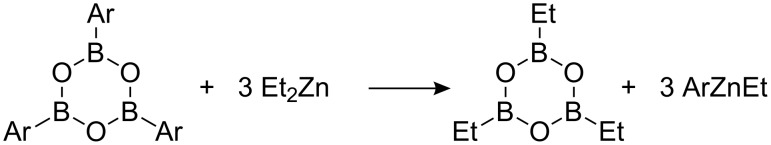
Generation of the mixed ArZnEt species from a triarylboroxin and Et_2_Zn.

On the other hand, triarylboroxins, easily prepared from the corresponding arylboronic acids by thermally induced dehydration under vacuum, have recently been applied with success as the starting materials for the preparation of the ArZnEt species to be used in the reaction [30–32] (Scheme 2). This represents a highly atom-economical approach since, in principle, no sacrificial excess of diethylzinc is needed for the transmetallation process and up to a 74% of the molecular mass of triphenylboroxin (the less favourable example) can be transferred to the reacting carbonyl compound.

It is to be mentioned that other strategies for the preparation of mixed alkylarylzinc species from cheap organometallic reagents have been developed in recent times and could probably be also used for the same purpose [33–35].

Flow chemistry [36–39] is increasingly seen as a promising methodology for the clean and economic production of complex substances. According to this, the field is experiencing a fast growth both in methodological aspects [40–43] and in applications [44–51]. In any case, examples of continuous flow enantioselective processes [52] are still scarce [22,53–57] in spite of the enormous potential of this methodology. Herein, we report the development of a continuous flow system for the preparation of enantioenriched diarylmethanols using triarylboroxins as the ultimate aryl group source.

## Results and Discussion

### Preparation of the immobilized catalyst

**Scheme 3:**
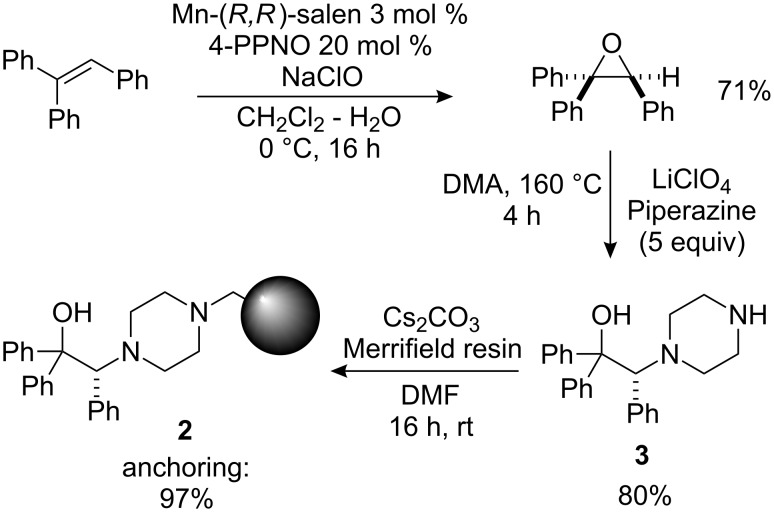
Synthesis of the immobilized amino alcohol **2**.

Resin **2** was prepared in three steps from commercially available triphenylethylene according to a reported procedure [21]. In the key steps, enantiomerically pure triphenylethylene oxide [58] is submitted to regioselective and stereospecific ring-opening with piperazine, and the resulting diamino alcohol **3** is subsequently supported onto a slightly cross-linked (1% DVB) Merrifield resin by direct treatment at room temperature in DMF in the presence of cesium carbonate (Scheme 3).

### Evaluation of resin **2** under batch conditions

**Scheme 4:**
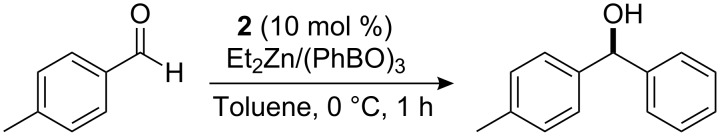
Phenylation of tolualdehyde catalyzed by **2**.

Prior to the continuous flow experiments, resin **2** was tested under batch conditions as a catalyst for the enantioselective phenylation of tolualdehyde using triphenylboroxin as the phenyl source (Scheme 4). In these studies, emphasis was put on the determination on the minimal ratio between triphenylboroxin and diethylzinc able to suppress competing ethyl transfer processes, and on the minimal amount of arylating species leading to complete conversion of the starting aldehyde.

Employing with **2** the reaction conditions previously optimized for the homogeneous ligand **1** [30], the addition product was obtained in slightly lower yield and enantioselectivity (entry 1, Table 1). This is probably due to the fact that the triethylboroxin co-product can trigger a non-enantioselective pathway for the arylation reaction; in fact, a decrease in the amount of (PhBO)_3_, as well as an increase in the amount of ZnEt_2_ employed for the transmetallation, allowed us to obtain the product in a somewhat better *ee*, although at the expense of a lower yield (entries 2 and 4). In the optimal conditions, when 2.5 equiv of ZnEt_2_ and 0.4 equiv of (PhBO)_3_ were used (entry 3), the diarylmethanol product was obtained in 73% yield and 89% *ee*.

**Table 1:** Optimization of batch conditions for the phenylation of tolualdehyde with triphenylboroxin with **2** as the catalyst.^a^

Entry	(PhBO)_3_ (equiv)	ZnEt_2_ (equiv)	Yield (%)	*ee* (%)

1	0.6	2.4	90	83
2	0.4	2.0	62	85
3	0.4	2.5	73	89
4	0.4	3.0	72	88

^a^All reactions run at 0 °C for 1 h at 0.2 M concentration of tolualdehyde.

### Continuous flow system

#### System set-up

**Figure 3:**
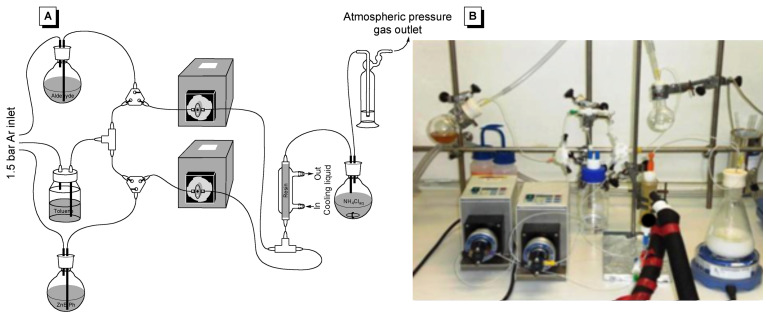
Experimental set-up for the continuous flow experiments. (A) Schematic representation and (B) Actual system.

For the continuous flow experiments, a system similar to that previously described for the enantioselective ethylation of aldehydes [23] was used (Figure 3). The flow reactor consists of a vertically mounted, fritted and jacketed low-pressure chromatography Omnifit glass column (10 mm bore size and a 70 mm maximal bed height) completely filled with the swollen resin. During operation, the reagents were pumped in through the bottom end of the column using two different piston pumps (one for the aldehyde substrate in toluene and one for the PhZnEt plus triethylboroxin mixture in toluene). Both flows were mixed in a T-shaped piece placed immediately before the reactor, in order to minimize the amount of background, non-enantioselective addition reaction before contact with the supported catalyst. Additionally, a supply of dry toluene was connected to both pumps, for swelling the resin and washing the tubing before and after the reaction. Isothermal operation was secured by circulation of a cooling fluid at the desired operation temperature through the column jacket. Finally, a collecting flask containing ammonium chloride solution was set in order to hydrolyze residual ethylating/arylating agents and thus prevent the non catalyzed reaction taking place in the event that conversion of the continuous flow catalytic reaction was not complete.

#### Optimization of the process

Optimization of reaction parameters under continuous flow conditions was performed again on the phenylation reaction of *p*-tolualdehyde. The results of this study are summarized in Table 2. Bearing in mind the strong acceleration usually observed when reactions are run under continuous flow conditions, due to the higher effective concentration of the catalyst, the use of a smaller excess of diethylzinc than under batch conditions was initially tested at 0 °C (entry 1). However, this resulted in low levels of conversion and enantioselectivity. As already observed under batch conditions, the use of a higher excess of diethylzinc led to increased conversion without loss of *ee* and no significant formation of side products (entry 2). On the other hand, increasing the reaction temperature to 20 °C led to an unacceptable decrease in enantioselectivity (entry 3). A further improvement of the performance of the system could be achieved by setting the reaction temperature to 10 °C (entries 4–6). Working at this temperature and adjusting the aldehyde:boroxin:diethylzinc ratio to 1:0.6:2.5 the product was obtained in 83% *ee*, with complete conversion and no significant decrease in yield due to the formation of byproducts (entry 6). It is worth mentioning that, in this way, the flow system could be operative for several hours without any significant degradation of the catalytic activity. For example, after 4 h, 3.2 g of (*S*)-phenyl(4-tolyl)methanol were obtained (80% yield) with 81% *ee*.

**Table 2:** Optimization of the reaction conditions for the continuous flow process.^a^

Entry	(PhBO)_3_ (equiv)	ZnEt_2_ (equiv)	T (°C)	Conv (%)^b^	Yield (%)^b^	*ee* (%)^c^

1	0.4	1.6	0	40	36	76
2	0.4	3.0	0	71	59	78
3	0.4	3.0	20	99	82	67
4	0.4	2.5	10	84	72	81
5	0.5	2.7	10	95	83	83
6	0.6	2.5	10	99	91	83

^a^All the reactions were performed with 1.1 g of resin, 0.24 mL/min total flow rate and 0.55 M maximal concentration of (PhBO)_3_. ^b^Conversion and yield determined by GC with tridecane as internal standard. ^c^*ee* determined by HPLC with a chiral column (for details see Supporting Information File 1).

#### Scope of the continuous flow arylation

**Scheme 5:**
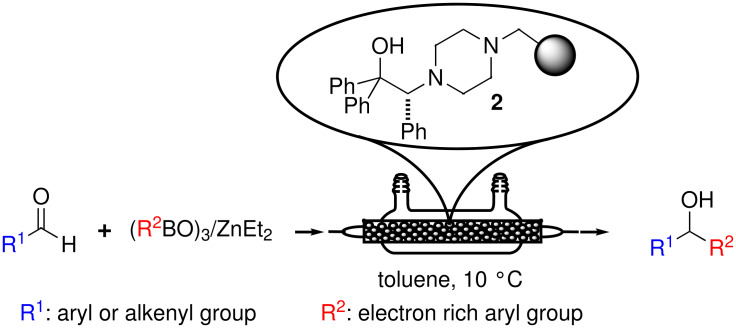
Continuous flow enantioselective preparation of diarylmethanols.

As previously demonstrated for catalyst **1**, this method can be used for the preparation of diarylmethanols with a wide variety of substituents, regardless of their electronic properties, provided that an adequate combination of aldehyde and arylating agent is chosen [30]. Thus, aryl(phenyl)methanols, with the aryl group bearing electron-withdrawing substituents, can be prepared easily by phenylation of the corresponding aldehyde. On the other hand, if a diarylcarbinol has to be prepared where one of the aryl groups bears electron donating substituents, the phenylation of the electron rich benzaldehyde is not efficient, so that the use of an electron rich boroxin in combination with benzaldehyde is preferred (Scheme 5).

We have summarized in Table 3 the results of the continuous flow arylation of a family of aldehydes. This study was done under the set of experimental conditions previously optimized for the phenylation of *p*-tolualdehyde (see Table 2, above), and the results given in the table refer to instant conversion and enantioselectivity after given reaction times.

**Table 3:** Substrate scope in the continuous flow arylation of aldehydes.^a^

Entry	Product	Time (h)	Conv (%)	% *ee*

1	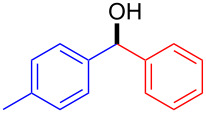	1 2 3 4 Overall^b^	99 98 98 98 80^d^ (76)^e^	83 82 81 79 81 (93)^e^
2	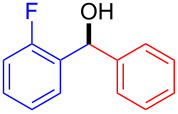	1 2 3 Overall^b^	99 99 99 91^d^	58 56 53 55
3	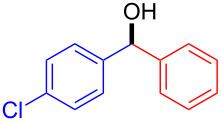	1 2 3 4 Overall^b^	99 99 99 99 78^d^ (67)^e^	72 70 67 65 68 (86)^e^
4	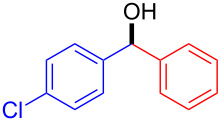	1 2 3 4 Overall^b^	>99 >99 >99 >99 93^d^	81 74 70 69 70
5	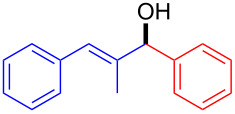	1 2 3 Overall^b^	99^c^ >99^c^ 82^c^ 82^d^	– – – 66
6	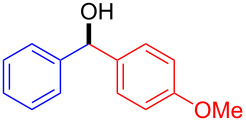	1 2 3 4 Overall^b^	99 >99 >99 97 83^d^	57 60 62 67 63

^a^Reaction conditions as in Table 2. For determination of conversion and *ee* see Supporting Information File 1. ^b^Data for the whole flow experiment. ^c^Determined by ^1^H NMR. ^d^Isolated yield. ^e^After a single recrystallization.

In all the studied cases tested, except in the phenylation of α-methylcinnamaldehyde (entry 5), the reaction could be run for some hours without significant decrease in the conversion of the starting aldehydes, thus allowing the preparation of enantioenriched carbinol products in multigram scale. Most attention was devoted to the use of PhZnEt (from triphenylboroxin and diethylzinc) in combination with different aromatic aldehydes (entries 1–4). Among these cases, the best results were obtained with *p*-tolualdehyde (entry 1). With *o*-fluorobenzaldehyde (entry 2), *p*-chlorobenzaldehyde (entry 3) and 2-naphthaldehyde (entry 4) conversions were excellent over the whole flow experiments (3 or 4 h), although enantioselectivities were slightly lower [30]. When an α,β-unsaturated aldehyde, such as α-methylcinnamaldehyde was used (entry 5), a fast reaction took initially place, although conversion was observed to slowly decrease after 2 h. Finally, (4-MeOC_6_H_4_)ZnEt [from tri(*p*-methoxyphenyl)boroxin and diethylzinc] could also be used in the continuous flow process with excellent conversion and moderate enantioselectivity (entry 6).

It is interesting to note that, although enantioselectivities were not as high as those obtained with the homogeneous catalyst **1** under batch conditions with the same arylating agents [30], or even with the heterogeneous catalyst **2** used under batch conditions with PhZnEt generated from diethylzinc and the very expensive diphenylzinc [22], the addition products could be easily crystallized in order to improve their enantiomeric purities. For instance, phenyl(4-tolyl)methanol could be obtained in pure form, in 76% yield and 93% *ee* after a single crystallization and 4-chlorophenyl(phenyl)methanol in 67% yield and 86% *ee* after the same process.

## Conclusion

In summary, the first single-pass, continuous flow enantioselective arylation of aldehydes has been developed. In this manner, enantioenriched diarylmethanols can be prepared in large scale through a simple and efficient process. The system has been optimized for the use of arylboroxins as an atom economical, cheap and readily available source of aryl groups. The simple procedures required for the purification and enantioenrichment of the resulting carbinols converts this flow process into a convenient alternative for the multigram production of these compounds.

The observed decrease in the enantioselectivity induced by the catalyst in comparison to its homogeneous analogue **1**, suggests some participation of triethylboroxin in a competing, non-enantioselective catalytic event. In fact, boroxins present adjacent atoms with complementary Lewis base (O) and Lewis acid (B) character that could coordinate the reactant molecules (aldehyde and arylethylzinc) in an arrangement suitable for reaction. In this sense, it is noteworthy that when control experiments were done by using resin **2** under batch conditions with the Ph_2_Zn/Et_2_Zn combination of reagents for the generation of PhZnEt, the observed enantioselectivities were comparable to those recorded with the homogeneous catalyst **1**. Thus, further improvement of the present continuous flow system could possibly be achieved with the use of alternative sources of the arylating species [33–35].

## Experimental

### General procedure for the arylation of aldehydes under batch conditions

A solution of ZnEt_2_ (257 mg, 2.08 mmol) in dry toluene (1 mL) was added via cannula to a suspension of phenylboroxin (104 mg, 0.333 mmol) in toluene (1 mL). The mixture was immediately warmed up to 60 °C in a preheated bath, in a closed system, and stirred in these conditions for 30 min before it was allowed to cool down to room temperature.

Meanwhile, catalyst **2** (*f* = 0.467 mmol/g, 180 mg, 0.083 mmol) was swollen with toluene (2 mL) for 30 min and then cooled to 0 °C. The ZnEt_2_/(PhBO)_3_ mixture was added and the resulting suspension was stirred for further 30 min.

After this time, tolualdehyde (100 mg, 0.83 mmol) was added and the reaction mixture was stirred at the same temperature for 1 h. Then, a saturated aqueous solution of NH_4_Cl was added to stop the reaction. The resin was removed by filtration and the biphasic mixture was diluted with dichloromethane and separated. The aqueous layer was extracted with dichloromethane and the combined organic layers were washed with aqueous NaHCO_3_ solution, dried with Na_2_SO_4_, filtered and concentrated under reduced pressure to obtain the product as a yellow oil.

Flash chromatography through silica gel with hexane-ethyl acetate mixtures afforded the pure phenyl(4-tolyl)methanol in pure form as a white solid (119 mg, 72% yield, 89% *ee*).

Conversion and GC yield were determined by GC analysis of samples of the organic solution before it was concentrated, with a HP-5 column. Enantiomeric excess was determined by HPLC analysis of the pure product with an AD-H column. Exact conditions for this and other products are given in Supporting Information File 1.

### General procedure for the phenylation of tolualdehyde in continuous flow conditions

The continuous flow system was set up as described in Figure 3. The column was filled with 1.1 g of resin, and it was swollen with a 0.24 mL·min^−1^ flow of dry toluene for 30 min. After that, a 1.1 M solution of the arylating agent, prepared as described above with (PhBO)_3_ (3.77 g, 12.1 mmol) and Et_2_Zn (6.23 g, 50.4 mmol) in toluene (33 mL), was connected to one of the pumps, at 0.12 mL·min^−1^, while toluene (0.12 mL·min^−1^) was kept in the other one, and the column was cooled down to 10 °C. These conditions were kept for a further 1 h, in order to form the amino alcohol-Zn complex. Then, a 0.61 M solution of tolualdehyde (2.42 g, 20.16 mmol) in toluene (33 mL) was connected to the second pump, keeping the same flow.

The product eluting of the column was collected in a flask containing a vigorously stirred aqueous NH_4_Cl solution, in order to stop the reaction.

The reaction progress was monitored by taking samples of approximately 50 μL and analyzing them by GC. The samples were treated with aqueous NH_4_Cl, extracted with dichloromethane and filtered through Na_2_SO_4_ before they were injected in the GC apparatus. These same samples were analyzed by HPLC in order to determine the *ee* (see Supporting Information File 1 for further detail).

After 4 h, both reactants were stopped and the system was washed by pumping toluene again for 30 min. The biphasic mixture containing the product was extracted with dichloromethane, washed with aqueous NaHCO_3_, dried with Na_2_SO_4_ and concentrated under reduced pressure. After flash chromatography on silica gel, 3.2 g of the product (80% yield) was obtained as a white solid, with 81% *ee*. A single crystallization from hexane afforded 2.4 g (76% yield) with 93% *ee*.

## Supporting Information

Supporting information contains GC and HPLC conditions for the analysis of the diarylmethanol products.

File 1 Conditions for the analysis of the diarylmethanols by GC and HPLC.
