# Supplementary material for: Continuous flow enantioselective arylation of aldehydes with ArZnEt using triarylboroxins as the ultimate source of aryl groups
Source: Beilstein J Org Chem. 2009 Oct 15;5:56. doi: 10.3762/bjoc.5.56 (PMC2813714; doi:10.3762/bjoc.5.56)
Supplement: File 1 — Conditions for the analysis of the diarylmethanols by GC and HPLC. [file Beilstein_J_Org_Chem-05-56-s001.pdf]

Continuous flow enantioselective arylation of aldehydes with ArZnEt using triarylboroxins as the ultimate source of aryl groups

Julien Rolland[1], Xacobe C. Cambeiro[1], Carles Rodríguez-Escrich[1] and Miquel A. Pericàs*[,1,2]

Address: [1]Institute of Chemical Research of Catalonia; Avinguda Països Catalans, 16; 43007 Tarragona, Spain and [2]Departament de Química Orgànica, Universitat de Barcelona; 08028 Barcelona, Spain.

Email: Miquel A. Pericàs - mapericas@iciq.es

* Corresponding author

Conditions for the analysis of diarylmethanols by GC and HPLC

General information:

The GC analyses were performed in an Agilent 6890N GC apparatus, with an Agilent HP-5 column and a FID detector. In every case, 1 μL of an approximately 15 μM solution of the product was injected. The injector was heated at 250 °C, and the mobile phase was He at 12 psi constant pressure, with a 25:1 split ratio. The detector was heated at 300 °C, with a 35 mL/min flow of H_2 and 350 mL/min of air.

The HPLC analyses were done in Agilent 1100 and 1200 series HPLC apparatus.

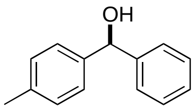

(*S*)-phenyl(*p*-tolyl)methanol:

GC: 70 to 115 °C (15 °C/min), then to 280 °C (25 °C/min), hold 0.4 min. R_t = 3.93 min (aldehyde), 5.54 min (tridecane), 8.29 min (alcohol).

HPLC: Chiralpak AD-H column, hexane–IPA 98:2, 1 mL/min, 210 nm. R_t = 28.0 min (*R*), 30.4 min (*S*).

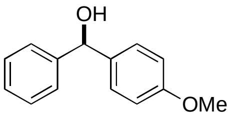

(*R*)-(4-methoxyphenyl)(phenyl)methanol:

GC: 70 to 115 °C (15 °C/min), then to 280 °C (25 °C/min), hold 0.4 min. R_t = 2.96 min (aldehyde), 5.54 min (tridecane), 9.05 min (alcohol).

HPLC: Chiralpak OJ column, hexane–IPA 95:5, 1 mL/min, 230 nm. R_t = 60.7 min (*R*), 69.4 min (*S*).

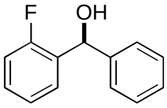

(*S*)-(2-fluorophenyl)(phenyl)methanol:

GC: 70 to 115 °C (15 °C/min), then to 280 °C (25 °C/min), hold 0.4 min. R_t = 2.84 min (aldehyde), 5.54 min (tridecane), 7.67 min (alcohol).

HPLC: Chiralpak AD-H column, hexane–IPA 98:2, 1 mL/min, 230 nm. R_t = 26.7 min (*S*), 28.5 min (*R*).

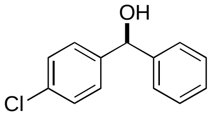

(*S*)-(4-chlorophenyl)(phenyl)methanol:

GC: 70 to 115 °C (15 °C/min), then to 280 °C (25 °C/min), hold 0.4 min. R_t = 4.30 min (aldehyde), 5.54 min (tridecane), 8.81 min (alcohol).

HPLC: Chiralpak AD-H column, hexane–IPA 95:5, 1 mL/min, 230 nm. R_t = 14.0 min (*R*), 15.4 min (*S*).

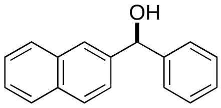

(*S*)-naphthalen-2-yl(phenyl)methanol:

GC: 70 to 115 °C (15 °C/min), then to 310 °C (25 °C/min), hold 4.2 min. R_t = 5.54 min (tridecane), 7.04 min (aldehyde), 10.50 min (alcohol).

HPLC: Chiralpak OD column, hexane–IPA 90:10, 1 mL/min, 230 nm. R_t = 13.1 min (*S*), 15.6 min (*R*).

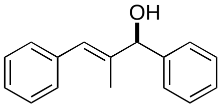

(*R,E*)-2-methyl-1,3-diphenylprop-2-en-1-ol:

HPLC: Chiralpak AD-H column, hexane–IPA 98:2, 0.5 mL/min, 254 nm. R_t = 42.1 min (*R*), 43.9 min (*S*).
